# Support for Sustainable Use of Personal Health Records: Understanding the Needs of Users as a First Step Towards Patient-Driven Mobile Health

**DOI:** 10.2196/mhealth.6021

**Published:** 2017-02-23

**Authors:** Se Young Jung, Keehyuck Lee, Hee Hwang, Sooyoung Yoo, Hyun Young Baek, Jeehyoung Kim

**Affiliations:** ^1^ Department of Family Medicine Seoul National University Bundang Hospital Seongnam Republic Of Korea; ^2^ Department of Pediatrics Seoul National University Bundang Hospital Seongnam Republic Of Korea; ^3^ Center for Medical Informatics Seoul National University Bundang Hospital Seongnam Republic Of Korea; ^4^ Department of Orthopedic Surgery Seoul Sacred Heart General Hospital Seoul Republic Of Korea

**Keywords:** electronic health record, medical informatics, personal health record, hospital information systems

## Abstract

**Background:**

The tethering of a personal health record (PHR) to an electronic medical record (EMR) may serve as a catalyst in accelerating the distribution of integrated PHRs. Creating shared health records for patients and their health care professionals using self-administered functions of EMR-tethered PHRs is crucial to support sustainable use of the system.

**Objective:**

This study assesses the factors related to active use of a self-administered function (Health Notes) in an EMR-tethered PHR (Health4U) in a tertiary academic hospital.

**Methods:**

This research is a cross-sectional study conducted in a tertiary academic hospital in South Korea. The enrollees included adults aged 19 years and older with experience accessing Health4U in the 13-month period after June 2013. The primary outcome was the adoption of Health Notes in accordance with the number of chronic diseases. Socio-demographic variables were included as confounding factors.

**Results:**

Subjects 71 years of age and older were less likely to become active users of Health Notes than those 30 years and younger. Moreover, compared with men, women had 44% and 40% lower tendencies to become Health Notes users and active users, respectively. Those who accessed the desktop page and/or mobile page had higher tendencies to become users of Health Notes. We found a consistent increase in the odds ratio as the number of chronic diseases increased in the active users. When considering specific diseases, patients who had cancer or chronic kidney disease had higher tendencies to become users of Health Notes.

**Conclusions:**

Patients with a greater number of chronic diseases tended to use PHR more actively, and used the self-administered function. Women and the elderly may have lower tendencies to actively use PHR. Therefore, items specific to the health of each demographic—women, the elderly, and those with chronic diseases—should be carefully considered to support sustainable use of PHRs.

## Introduction

Google Health was discontinued in January 2012, less than 3 years after its inception [1,2]. Google’s original intent was to disseminate consumer-centric values that it had successfully established in other areas within the field of health care by providing users with an opportunity to access all personal health records (PHRs) and useful health information. However, Google soon realized that, in contrast to its initial expectation, people who used the service were limited to a small number of users with interests in information technologies [1]. Google's candid confession clearly suggested the limitations of a standalone PHR.

In a symposium organized by the American Medical Informatics Association’s College of Medical Informatics in 2005, participants concluded that an electronic medical record (EMR)-tethered PHR can provide greater value than a standalone PHR [3]. A standalone PHR presents many challenges, particularly with information accountability; as information entry is solely dependent on the users’ ability to periodically update their information, failure to do so will likely be ineffective [3,4]. Conversely, if PHRs were connected to EMRs, patients would have the benefit of being able to take advantage of a system that automatically generated their personal health information during their visitation to the hospital via the connected Hospital Information System. Compared with a standalone PHR, higher-quality and objective information can be provided by an EMR-tethered PHR. In addition, this type of system can process hospital data in diverse formats and can provide the data directly to the patients [5]. However, patients may lack initiative to manage their medical information actively if they are only given information that is automatically generated by the EMR, and if they are not provided with perceptive value to use PHRs [3]. Therefore, EMR-tethered PHRs should offer a convenient way for both patients and physicians to create a shared records database and provide self-administered features, which may be the first step in supporting the sustainable use of PHRs, in terms of providing patients with consumer-centric values [6,7].

Although EMR-tethered PHRs have been attempted in many medical institutions, only a few studies have been conducted on how the system can be served to improve self-administered functions. We can overcome the shortcomings of EMR-tethered PHRs by analyzing the gap between patients’ needs and self-administered functions, in an effort to sustain users’ interest.

This study explored the features of EMR-tethered PHRs used in Seoul National University Bundang Hospital (SNUBH) in South Korea, and investigated the demographics of the frequent users of the self-administered features in EMR-tethered PHRs. Based on the findings, this study also suggests additional functions that can be incorporated into the system of EMR-tethered PHRs in the future.

## Methods

### Development Process of Electronic Medical Record-Tethered Personal Health Records

A task-force team was established to conduct a needs-analysis and develop PHRs with the name of *Health4U*. Health4U was established based on the needs of users, mainly composed of five parts: visit history, prescription history, drug notification, laboratory results, and management of self-administered component (called the *Health Notes*). Patients can record their daily blood pressure, blood sugar, amount of exercise, and body weight in the Health Notes [8].

### Study Population

**Figure 1:**
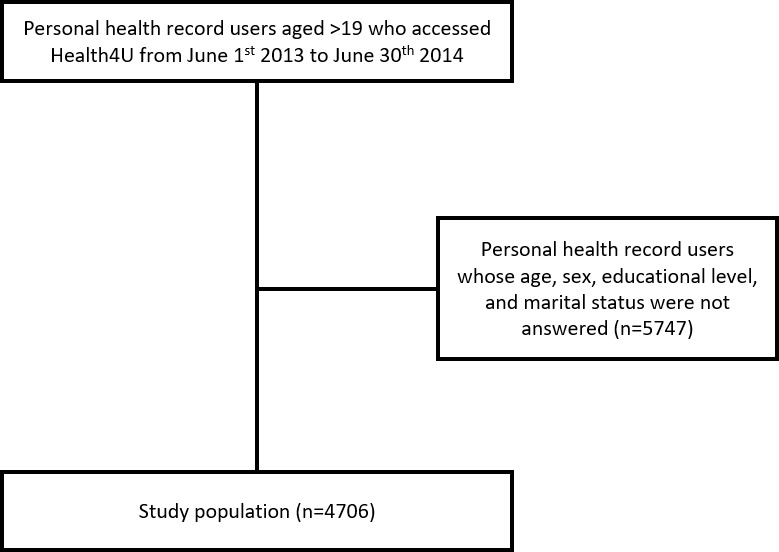
Study population.

This study used cross-sectional data extracted from a clinical data warehouse of SNUBH. The enrollees were selected from adults aged 19 years and older with prior experience accessing Health4U in the 13-month period after June 2013, when the service was first initiated. A total of 4706 users of Health4U were included in this study (Figure 1).

### Observation and Statistical Analysis Method

The following socio-demographic variables were included: age, gender, educational level, marital status, religion, method used to access Health4U, and main services utilized. Age was divided into 10-year increments for comparison. Educational level was categorized into three groups: middle school and lower, high school degree, and college degree and above. The access method was also divided into three groups: access by mobile application only (mobile-only group), access by personal computer website only (desktop-only group), and access with both (desktop-mobile group). The services used for the analysis included the number of views for treatment history, prescription information, medication reminders, test results, and Health Notes.

Users of Health4u were defined as individuals who accessed the system once or more. Users of Health Notes were defined as those who used the feature once or more, and nonusers were defined as those who did not use Health4U. Active users of Health Notes were defined as those who used the feature three times or more. The factors associated with becoming a Health Notes user or active user were investigated using univariate analyses.

Diseases that the Health Notes users had can be considered as important factors for improving the self-administered features of EMR-tethered PHRs. In this study, diabetes, hypertension, dyslipidemia, obesity, and chronic kidney disease were included as representative chronic diseases, and acute coronary syndrome was included as a representative acute disease.

A multivariate logistic regression analysis was performed to adjust for potential confounding factors. In the first model, an analysis was conducted using the number of chronic diseases that showed a *P*-value of less than 0.25 as a covariate in a univariate analysis. In the second model, an analysis was performed using each disease as a covariate. *P*-values of less than 0.05 were considered statistically significant, and Stata 13.0 (Stata Corp., College Station, TX, USA) was used for statistical analyses. This study was approved by the Institutional Review Board at the SNUBH. The requirement of informed consent was waived because we used nonidentiﬁed retrospective data.

## Results

### Use of Health4U

Among the 4706 users of Health4U included in this study, 373 users accessed both the mobile application and the website, while 2459 users accessed the mobile application only, and 1874 users accessed the website only. The age groups were distributed between 10-21%. Men used Health4U more than women (2444/4706, 51.93%; Table 1).

**Table 1:** Baseline characteristics.

Characteristics		Number	Percentage
Age			
	19-30	500	10.62
	31-40	974	20.70
	41-50	989	21.02
	51-60	965	20.51
	61-70	654	13.90
	71 or more	624	13.26
Gender			
	Male	2444	51.93
	Female	2262	48.07
Education level			
	Middle school and lower	330	7.01
	High school degree	1489	31.64
	College degree and above	2887	61.35
Having spouse			
	No	864	18.36
	Yes	3842	81.64
Modes of access			
	Mobile only	2459	52.25
	Desktop only	1874	39.82
	Both	373	7.93
Components (mean)			
	View visit history	6.17	
	View prescription history	2.39	
	View drug notification	3.47	
	View laboratory result	14.15	
	View Health Notes	5.43	

The function most commonly utilized by the users was to view test results, with an average of 14.15 views. The need for viewing Health Notes was third most commonly used, with an average of 5.43 views.

### Self-Administered Functions of Health4U (Health Notes)

**Figure 2:**
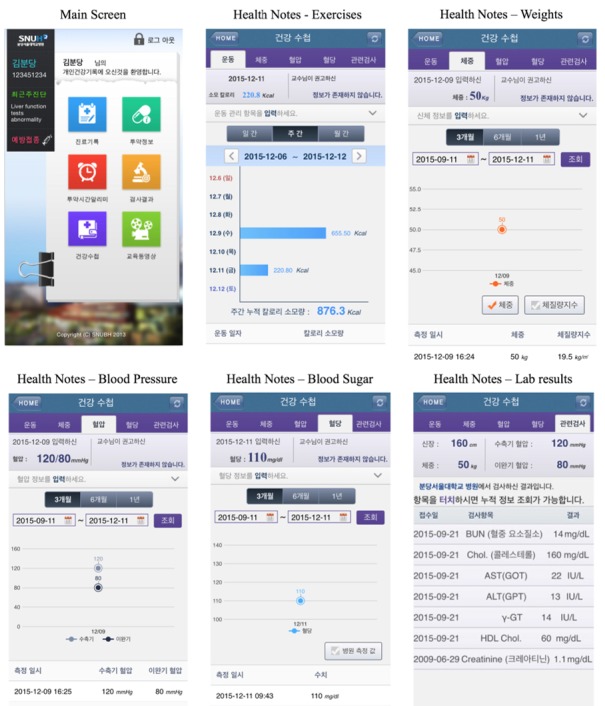
Main screen of mobile Health4U, and five key components of Health Notes: amount of exercise, weight and height, blood pressure with measurement time, blood sugar with measurement time, and laboratory results relevant to users’ current medical status.

Health Notes were developed to include five main components (Figure 2). Users can input their daily amount of exercise to compare it to their doctor’s recommendations. Amounts of exercise can be monitored weekly and monthly, and users can manage their daily weight, height, blood pressure, and blood sugar. Additionally, users can monitor these data every 3, 6, and 12 months, and can also see their laboratory results related to their diseases.

### Analysis of the Health Notes Completion Traits

Both users and active users of Health Notes were an average of >3 years younger than nonusers and nonactive users, respectively. The age group of 61 years and older had a lower tendency to become users of Health Notes compared to the 19-30-year age group. Women completed Health Notes 50% less frequently than men, and the group with an educational level of college degree and above completed Health Notes more than the group with middle school education or below. The desktop-only group and the desktop-mobile group had higher tendencies to become users of Health Notes, compared with the mobile-only group.

Regarding disease association, those who had diabetes, dyslipidemia, cancer, obesity, chronic kidney disease, or acute coronary syndrome had higher tendencies to become users or active users of Health Notes. Those who had a greater number of chronic diseases had higher tendencies to become users and active users of Health Notes (Multimedia Appendix 1).

After adjusting for covariates in the first model, the age group of 61 years and older had a lower tendency to become users of Health Notes; the age group of 71 years and older had a lower tendency to become active users than the age group of 30 years and younger. Compared with men, women had 44% and 40% lower tendencies to become Health Notes users and active users, respectively. The desktop-only group or desktop-mobile group had higher tendencies to become users of Health Notes, and the desktop-mobile group had a higher tendency to become active users than the mobile-only group. Although we found a consistent increase of odds ratios as the number of chronic diseases of Health Notes users increased, the results showed a statistical significance when they had 1 chronic disease or 3 chronic diseases. Regarding active users, we found a consistent increase of odds ratios as the number of chronic diseases increased, with statistical significance when they had 1 or more chronic diseases (Table 2).

**Table 2:** Multivariable analysis of the factors associated with completing Health Notes in Health4U (Model 1).

Variable		Health Notes Usage		Health Notes Active Usage	
		Adjusted Odds Ratio (95% CI)	*P*-Value	Adjusted Odds Ratio (95% CI)	*P*-Value
Age					
	19-30	1		1	
	31-40	0.91	.71	0.80	.54
	41-50	0.92	.78	0.83	.62
	51-60	0.82	.51	0.99	.99
	61-70	0.43	.018	0.54	.18
	71 or more	0.24	.001	0.28	.02
Gender					
	Male	1		1	
	Female	0.56	<.001	0.60	.009
Education level					
	Middle school and lower	1		1	
	High school degree	1.22	.66	1.66	.50
	College degree and above	1.35	.51	1.65	.48
Having spouse					
	No	1		1	
	Yes	0.91	.64	0.67	.14
Modes of access					
	Mobile only	1		1	
	Desktop only	1.66	.002	1.29	.25
	Both	7.01	<.001	5.94	<.001
Number of chronic diseases					
	0	1		1	
	1	1.68	.007	2.41	<.001
	2	1.72	.065	2.77	.003
	3	6.83	<.001	6.99	.001
	4	8.78	.164	1	Not applicable

In the second model, it was found that the difference in age, gender, and method of access was similar to that of the first model; the group with cancer and chronic kidney disease had higher tendencies to become Health Notes users or active users (Table 3).

**Table 3:** Multivariable analysis of the factors associated with completing the health notes in Health4U (Model 2).

Variable		Health Notes Usage		Health Notes Active Usage	
		Adjusted Odds Ratio (95% CI)	*P*-Value	Adjusted Odds Ratio (95% CI)	*P*-Value
Age					
	19-30	1		1	
	31-40	0.88	.64	0.77	.45
	41-50	0.90	.72	0.84	.64
	51-60	0.80	.45	0.980	.96
	61-70	0.40	.011	0.53	.16
	71 or more	0.23	.001	0.24	.012
Gender					
	Male	1		1	
	Female	0.56	<.001	0.58	.008
Education level					
	Middle school and lower	1		1	
	High school degree	1.29	.58	1.89	.32
	College degree and above	1.42	.44	1.80	.35
Having spouse					
	No	1		1	
	Yes	1.12	.58	1.51	.13
Modes of access					
	Mobile only	1		1	
	Desktop only	1.63	.003	1.32	.21
	Both	6.58	<.001	5.45	<.001
Diabetes					
	No	1		1	
	Yes	1.24	.347	1.39	.25
Dyslipidemia					
	No	1		1	
	Yes	1.36	.19	1.58	.12
Cancer					
	No	1		1	
	Yes	1.38	.031	1.57	.021
Obesity					
	No	1		1	
	Yes	2.11	.072	1.77	.29
Chronic Kidney Disease					
	No	1		1	
	Yes	2.75	.001	4.00	<.001
Acute Coronary Syndrome					
	No	1		1	
	Yes	1.39	.24	1.26	.53

## Discussion

In this study, we have revealed a significant association between the use of a self-administered function of an EMR-tethered PHR and the number of chronic diseases the users had. Regarding specific diseases, patients who had cancer or chronic kidney disease had higher tendencies to become users or active users of Health Notes. Additionally, we found that those who were 61 years and older had a lower tendency to become Health Notes users compared to those who were 30 and younger. Men were more likely to become Health Notes users than women, and those who accessed the desktop page were more likely to become Health Notes active users compared to those who only accessed the mobile page.

### Differences in Health Notes Completion by Age

Our findings, with respect to the generation gap in the use of Health Notes, were similar to a previous study [9,10]. Accordingly, older adults lacking experience with technology encountered greater problems using PHRs [9,10]. Elderly people tend to have lower income and lower literacy for new technology compared to younger people, as indicated by previous studies [11,12]. A previous study also revealed that low-income elderly would not receive benefits from PHRs due to poor technical skills, low literacy, and limited cognitive/physical ability [13,14]. Due to the differences in PHR usage by age, which can lead to health inequality between generations, a feature must be developed to enhance the accessibility and usability of Health Notes for older adults.

### Differences in Health Notes Completion by Gender

Women had a lower tendency to become Health Notes users or active users. A report by the Broadband Commission Working Group revealed that there exists a gap in the use of information technology between men and women, and that approximately 200 million fewer women (compared to men) access information technology on the Internet globally [15]. Therefore, items specific to women’s health, or efforts to promote campaigns that target women, should be developed to overcome gender differences in Health Notes usage. PHR functions for health care during pregnancy and postmenopausal periods are viable options.

### Differences in Health Notes Completion by Method of Access

The group that used Web-based PHRs tended to be active users of Health Notes, highlighting the importance of allowing users to easily administer their own health information. Most of the items that can be entered into Health Notes require measurement equipment, such as individual physical measurements, as well as blood pressure and blood sugar. Such equipment is often placed in the vicinity of a personal computer (in a home or office), making it more likely that the resulting information is entered by accessing the Web-based PHR; after measuring their values, it is relatively easy for patients to enter this information directly into the PHR system. When using the mobile PHRs, health information must be entered using a smartphone’s virtual keyboard. Using this method, information can be entered from anywhere, especially when personal computers are not an option.

### Differences in Health Notes Completion by the Presence of Chronic Diseases

In the first model, we found that those who had more chronic diseases tended to become active users of Health Notes. This study indicates that patients with chronic diseases have a higher desire to use Health Notes. However, a previous study revealed that it has remained impossible to conclude that the use of PHRs can be effective for improving chronic diseases [16].

In addition, a previous study on the use of the Internet in diabetes management suggested that the frequency of website use for diabetes management decreased over time [17]. Taken together, although patients with a chronic disease tend to actively use PHRs, it is insufficient to assert that using such a feature (when installed in an existing PHR) can translate to significant improvements in health outcomes. Patients with chronic diseases may also encounter barriers to the continual use of PHRs. One solution to this problem might be to provide patients with easy opportunities to visualize how the management of their blood pressure, body weight, and blood sugar can affect their chronic disease by relying on more specific values (eg, cardiovascular risk scores) and providing these values to patients. For example, if diabetic patients are provided with their annual test results (including a retinal examination, microalbuminurial test, and renal function test), as well as imaging tests taken at the hospital (eg, carotid sonogram, coronary angiography, and brain magnetic resonance imaging/angiogram) and a comprehensive report, they may become more motivated to actively manage their health via PHRs.

In the second model, which analyzed each disease separately, patients with cancer or chronic kidney disease had higher tendencies to become users or active users of Health Notes. However, it was found that health diaries lacked a sufficient number of items to help cancer patients manage their health. One future option could be to implement a feature in which cancer patients under treatment can record their health conditions, or a feature that reminds cancer survivors that it is time for postcancer examinations. One study demonstrated that the rates of mammogram screening and flu vaccination increased when a reminder was provided via a standalone PHR for health maintenance [18]. Other insufficient items were observed for the management of chronic kidney disease. A feature that could inform the residual renal function would be helpful for sustaining the interest of patients with chronic kidney disease.

In 2010, the Obama administration rolled out a five-year plan for making doctors and hospitals move to electronic health records (EHRs), which are closely related to precision medicine and personalized medicine [19-21]. As of 2013, 78% of office-based doctors used some form of EHR system, up from 18% in the United States in 2001 [22]. The transition to EHRs has augmented the scope of medical record-based information [23,24]. However, quantitative development has not guaranteed qualitative improvement because the quality of the data entered remained unchanged [20]. PHR development and adoption can hasten EHR distribution and upgrade the quality of EHR by providing crucial values to patients, physicians, and health care providers. The goal of these efforts is to provide patient-centered, timely, and efficient health care. A previous study showed that creating shared health records for patients and their health care professionals can improve patients’ ability to become active partners in their own health care [6]. Another study showed that patients wanted to improve the doctor-patient relationship by actively using PHRs [25]. However, thus far, PHRs themselves are facing a huge barrier to continuous development [5,26,27]. There have been many studies conducted to improve PHRs [16,18,28,29], yet only a few studies have been conducted in which patients used the self-administered features of EMR-tethered PHRs, which can enable shared health care and patient-centered practice. If we fail to understand the needs of PHR users, PHRs would inevitably fail to satisfy the users’ needs. As a first step to move from rudimentary standalone PHRs to integrated PHRs, EMR-tethered PHRs can offer clues about how we can improve PHRs by implementing patient-centric features in the system.

As the first hospital to attain Healthcare Information and Management System Society Stage 7 status outside of North America, SNUBH introduced a comprehensive EHR to all divisions of the hospital in 2003, launching a connected PHR service in 2013 [30]. Through this study, based on this EHR-friendly circumstance, we have suggested for the first time that users with more chronic diseases tend to use PHR more actively, and regularly utilize the self-administered function. This finding can play a crucial role in developing future functions of PHRs.

### Future Directions

First, PHRs must integrate a feature that enhances the accessibility and usability of the self-administered function for older adults. Second, items specific to women’s health should be created to overcome the gender differences in PHR usage. Third, PHR functions for each chronic condition should be made to promote PHR usage for patients with chronic diseases. Finally, to maximize mobile device usage of self-administered functions, one solution would be to use a method that automatically transmits the data measured from a blood pressure monitor, a blood glucose monitor, or a body weight scale to the mobile device via Wi-Fi, without requiring the user to enter the information directly. The incorporation of Wi-Fi capabilities into medical devices could lead to reduced health care costs, while allowing medical teams to obtain patients’ health information in real time [31].

### Limitations

There are limitations in generalizing the results of this study, due to the fact that the study only involved one university hospital. However, because this study was focused on the use of EMR-tethered PHRs at a large hospital, where the use of EMRs has been in place for more than 10 years, these results will serve as important data for medical institutions that intend to develop the same features, or for national agencies planning to develop integrated PHRs.

This is a cross sectional study, making it difficult to find causal relationships, and the study lacks information on the precise improvement in the health outcomes of PHR users or those who completed Health Notes. This limitation should be offset by further studies. To examine the effects on health outcomes, an analysis is needed regarding the related diseases of those who actively used Health Notes in EMR-tethered PHRs, and the features of PHRs need to be expanded according to the diseases. Hence, this study is relatively significant as it presents the direction of PHR development for the future.

### Conclusion

This is the first study that discovered the factors related to the completion of a self-administered function of PHRs tethered to a comprehensive EHR, which can be considered as one of the important determinants of active use of PHRs. The finding that patients with more chronic diseases tended to be active users of PHRs can serve as the basic data for enhancing the features of an EMR-tethered PHR system in the future.

## Appendix

Multimedia Appendix 1 Univariate analysis of the factors associated with completing Health Notes in Health4U.

## Abbreviations

EHR: electronic health record
EMR: electronic medical record
PHR: personal health record
SNUBH: Seoul National University Bundang Hospital
