# Supplementary material for: Support for Sustainable Use of Personal Health Records: Understanding the Needs of Users as a First Step Towards Patient-Driven Mobile Health
Source: JMIR Mhealth Uhealth. 2017 Feb 23;5(2):e19. doi: 10.2196/mhealth.6021 (PMC5344982; doi:10.2196/mhealth.6021)
Supplement: Multimedia Appendix 1 [file mhealth_v5i2e19_app1.pdf]

Multimedia Appendix 1. Univariate analysis of the factors associated with completing the Health Notes in Health4U.

	Health Notes Users	Health Notes Non-	Odd Ratio	P value	Health Notes	Health Notes	Odd Rati	P value
Age (mean, SD)	50.46 (16.08)	46.14(13.29)			50.33 (16.03)	47.05 (13.69)		
19-30	30	470	1.00		18	482	1.00	
31-40	57	917	0.97	0.91	26	948	0.73	0.32
41-50	61	928	1.03	0.9	29	960	0.81	0.49
51-60	57	908	0.98	0.94	35	930	1.01	0.98
61-70	20	634	0.49	0.017	13	641	0.54	0.10
71 or more	12	612	0.31	0.001	7	617	0.30	0.10
Gender								
Male	160	2284	1.00		87	2357	1.00	
Female	77	2185	0.50	<0.001	41	2221	0.50	<0.001
Education level								

≤Middle school	6	324	1.00		3	327	1.00	
High school	60	1429	2.27	0.058	38	1451	2.85	0.08
≥College	171	2716	3.40	0.004	87	2800	3.39	0.04
Having spouse								
No	54	810	1.00		33	831	1.00	
Yes	183	3659	0.75	0.072	95	3747	0.64	0.29
Modes of access								
Mobile only	69	2390	1.00		41	2418	1.00	
Desktop only	96	1778	1.87	<0.001	47	1827	1.52	0.05
Both	72	301	8.29	<0.001	40	333	7.08	<0.001
Diabetes								
No	202	4023	1.00		105	4120	1.00	
Yes	35	446	1.56	0.019	23	458	1.97	0.00
Hypertension								

No	196	3817	1.00		107	3906	1.00	
Yes	41	652	1.22	0.25	21	672	1.14	0.59
Dyslipidemia								
No	200	4052	1.00		105	4147	1.00	
Yes	37	417	1.80	0.00	23	431	2.11	0.00
Cancer								
No	149	3081	1.00		77	3153	1.00	
Yes	88	1388	1.31	0.05	51	1425	1.47	0.04
Obesity								
No	228	4411	1.00		123	4516	1.00	
Yes	9	58	3.00	0.00	5	62	2.96	0.02
Chronic Kidney Disease								
No	219	4344	1.00		114	4449	1.00	
Yes	18	125	2.86	<0.001	14	129	4.24	<0.001

Acute Coronary Syndrome								
No	217	4240	1.00		117	4340	1.00	
Yes	20	229	1.71	0.03	11	238	1.71	0.09
Number of chronic diseases								
0	171	3668	1.00		83	3756	1.00	
1	42	585	1.54	0.015	29	598	2.19	<0.001
2	16	188	1.83	0.027	12	192	2.83	0.001
3	7	27	5.56	<0.001	4	30	6.03	0.001
4	1	1	21.45	0.03	0	2	1.00	NA
□	□	□	□	□	□	□	□	□

Abbreviation: NA, not available

Chronic diseases included in the number of chronic diseases: diabetes, dyslipidemia, obesity and chronic kidney disease, which showed a level of significance of less than 0.25

The active users of the Health Notes were defined as those who used it three times or more
